# Organizational inaction sustains destructive leadership in healthcare

**DOI:** 10.3389/fpsyg.2026.1859185

**Published:** 2026-09-14

**Authors:** Roland Seese, Katalin Dióssi

**Affiliations:** 1 Department of Economics and Management, FOM University of Applied Sciences for Economics and Management, Dortmund, Germany; 2 Alexandre Lamfalussy Faculty of Economics, University of Sopron, Sopron, Hungary

**Keywords:** destructive leadership, healthcare organizations, institutional decoupling, normalization of deviance, organizational inaction, organizational silence, psychological safety

## Abstract

Destructive leadership is increasingly recognized as a distinct and harmful form of influence, yet the response of organizations to such leadership remains theoretically underdeveloped. Objective: This article develops a theory of organizational inaction in the face of destructive leadership and explains why organizations differ in their capacity to recognize and confront such conduct. Using a theory-driven, integrative conceptual-development approach, this Hypothesis and Theory article synthesizes research on destructive leadership, organizational silence, psychological safety, institutional theory, normalization of deviance, and useful illegality to develop an explanatory framework of organizational inaction. Organizational inaction is defined as the systematic failure of an organization to recognize, process, escalate, or intervene against destructive leadership despite the availability of signals, experiences, or structural indicators that harmful leadership is occurring. The article differentiates this construct from organizational silence, psychological safety, institutional inertia, and the organizational bystander effect. Building on the healthcare sector as a particularly revealing organizational field, the article proposes that organizational response depends on two capacities: recognition and intervention. Crossing both dimensions results in four heuristic ideal types on continuous dimensions—functional, blind, paralyzed, and incapable organizations. The article further develops formal propositions that specify the antecedents, mechanisms, and consequences of these types and integrates organizational silence, psychological safety, institutional theory, and normalization of deviance into the proposed model. The article argues that destructive leadership persists not only because of leader pathology or follower susceptibility, but also because organizations vary systematically in the structures, interpretations, and informal practices that shape whether harmful leadership is seen and whether action becomes possible.

## 1 Introduction

For much of its history, leadership research concentrated on positive and effective forms of influence. Motivation, charisma, transformational leadership, ethics, and performance dominated the field, while harmful leadership was often treated as an anomaly or simply as the absence of good leadership. This imbalance has narrowed as destructive leadership has developed into a distinct field concerned with repeated leader behavior that harms employees and organizations (Thoroughgood et al., 2018; Schilling and Schyns, 2021). Recent systematic reviews and meta-analyses synthesize a sizeable evidence base on its effects (Montano et al., 2023; Negt, 2023).

This shift matters because the harm is substantial. Meta-analytic evidence links destructive leadership to affective symptoms, stress, burnout, health complaints, and reduced well-being (Montano et al., 2023; Negt, 2023). Longitudinal evidence also indicates that destructive leadership can erode employees' sense of meaningful work and their productivity over time (Grill, 2023). These consequences extend beyond the relationship between a leader and an individual follower and can impair organizational functioning.

Healthcare provides a particularly revealing context for this problem. Strong professional hierarchies, resource constraints, regulatory demands, and operational interdependence shape who can raise concerns and how those concerns are handled. A recent scoping review shows that hierarchy affects not only whether healthcare staff speak up, but also what can be said, by whom, and at what point (Essex et al., 2023). Research on employee voice likewise identifies organizational, leadership, team, and individual influences on speaking up in healthcare (Hague et al., 2025). At the same time, recent evidence links destructive or toxic leadership to burnout, workforce instability, nurse retention, care quality, and patient safety (Hult et al., 2023; Labrague, 2023; Palvimo et al., 2023; Tsapnidou et al., 2025).

This evidence establishes the harm associated with destructive leadership but leaves a different problem unresolved: what happens after warning signals emerge. Concerns may remain local because employees stay silent, may lose force as they move through hierarchical channels, or may reach formal reporting and governance structures without prompting action. Recent review evidence suggests that negative leadership is often associated with nurse silence, although the limited and heterogeneous evidence base does not support a simple universal relationship (Li et al., 2024). The problem addressed in this article is organizational: why do some organizations recognize and confront destructive leadership, while others fail to convert available signals into timely and credible intervention?

Existing leader-, follower-, and environment-centered models explain important antecedents and enabling conditions of destructive leadership (Padilla et al., 2007; Thoroughgood et al., 2018). Research on silence and voice explains why concerns may not be communicated, while institutional research shows why formal structures can remain disconnected from organizational practice. These literatures, however, are usually examined separately. They offer only a limited account of the complete organizational response process, especially the transition from local experience to organizational recognition and from recognition to intervention (Morrison, 2023; Hult et al., 2023; Hague et al., 2025). This is the research gap addressed here.

To address this gap, the article develops organizational inaction as a distinct organizational-level construct. It refers to the systematic failure to recognize, process, escalate, or intervene against destructive leadership despite the availability of relevant signals, experiences, or structural indicators. The framework distinguishes recognition capacity from intervention capacity and uses the two dimensions to derive four heuristic organizational types. It then links these types to organizational silence, psychological safety, institutional decoupling, normalization of deviance, organizational gravity, and useful illegality. The resulting propositions provide a basis for empirical examination in healthcare and other high-pressure organizational settings.

The argument develops from construct clarification to mechanism-based theory building. Organizational inaction is distinguished from related concepts and then connected to research on destructive leadership, organizational silence, psychological safety, institutional decoupling, normalization of deviance, organizational gravity, and useful illegality. Healthcare serves as a critical context in which tensions between recognition and intervention become especially visible under conditions of hierarchy, resource scarcity, and operational interdependence. Building on this synthesis, the article develops the proposed typology and seven propositions. The discussion considers the consequences of organizational inaction, its theoretical and practical implications, the limitations of the framework, and priorities for empirical research.

## 2 Conceptual development and analytical approach

This Hypothesis and Theory article uses an integrative conceptual-development approach (Jaakkola, 2020). Its aim is to build an explanatory framework for how organizations process signals of destructive leadership, not to estimate prevalence or effect sizes. The synthesis connects research on destructive leadership and its multilevel antecedents (Padilla et al., 2007; Thoroughgood et al., 2018; Schilling and Schyns, 2021), employee silence and voice (Morrison and Milliken, 2000; Morrison, 2023; Lainidi et al., 2023), psychological safety (Edmondson, 1999; Edmondson and Bransby, 2023), institutional decoupling (Meyer and Rowan, 1977; Mor Barak et al., 2022), normalization of deviance (Vaughan, 1999; Wright et al., 2022), organizational gravity (Schneider et al., 2000; Nerdinger, 2014b), and useful illegality (Kühl, 2019; Luhmann, 2022). These streams address different stages of the organizational response problem and are brought together here to explain variation in recognition and intervention.

The synthesis was developed iteratively and organized by explanatory relevance. Initial contributions were drawn from the focal theoretical traditions, followed by backward and forward citation searches. Targeted searches were then used to identify reviews and empirical studies published from 2020 onward on destructive leadership, silence and voice, psychological safety, decoupling, normalization of deviance, and healthcare. Sources were retained when they defined a focal construct, provided evidence about a mechanism affecting recognition or intervention, or clarified how the mechanisms could be combined in a typological model. Foundational works were retained where they established the construct, while recent sources were used to assess its continuing relevance. Because the search was selective and theory-led and did not use an exhaustive screening protocol, the article presents an integrative conceptual synthesis, not a systematic literature review (Whetten, 1989; Jaakkola, 2020).

The analytical sequence began by examining what leader-, follower-, and environment-centered accounts leave underdeveloped: how organizations process warning signals after harmful conduct emerges (Padilla et al., 2007; Thoroughgood et al., 2018; Hult et al., 2023). The analysis then differentiated organizational inaction from organizational silence, psychological safety, institutional inertia, and the organizational bystander effect (Morrison and Milliken, 2000; Hannan and Freeman, 1984; Darley and Latané, 1968). Recent research confirms that voice and silence are shaped by organizational and professional conditions, while psychological safety concerns the interpersonal risk attached to speaking up (Morrison, 2023; Edmondson and Bransby, 2023; Lainidi et al., 2023). Organizational inaction is broader because it also includes situations in which concerns are known but fail to produce escalation or intervention.

The identified mechanisms were classified by the stage of organizational response they most directly affect. Organizational silence and low psychological safety constrain recognition because they influence whether locally experienced harm is voiced, treated as credible, and incorporated into organizational knowledge (Morrison, 2023; Edmondson and Bransby, 2023; Lainidi et al., 2023). Institutional decoupling and useful illegality primarily constrain intervention: policies may exist and concerns may be formally acknowledged while everyday practices continue to protect operational priorities or informal arrangements (Meyer and Rowan, 1977; Mor Barak et al., 2022; Kühl, 2019; Luhmann, 2022). Normalization of deviance and organizational gravity operate across both stages. Repeated deviations alter what is regarded as normal, while organizational selection and retention processes affect which behavioral profiles become dominant and which conduct is seen as problematic or sanctionable (Vaughan, 1999; Schneider et al., 2000; Nerdinger, 2014b; Wright et al., 2022). This classification produced the distinction between recognition capacity and intervention capacity as separate but interdependent continuous dimensions.

Crossing the two dimensions produced four heuristic ideal types: functional, blind, paralyzed, and incapable organizations. In accordance with ideal-type and typological theory building, these configurations are intended as analytical simplifications and not as empirically discrete categories or validated diagnostic classes (Weber, 1949; Doty and Glick, 1994; Jaakkola, 2020). The typology was then connected back to the identified mechanisms in order to formulate propositions concerning the antecedents of recognition and intervention deficits and the possible movement between organizational types. The propositions translate the conceptual relationships into claims that can be examined empirically, while Table 1 and Supplementary Table 1 specify how such examination may be conducted through comparative qualitative interviews, process reconstruction, negative-case analysis, and the consideration of plausible rival explanations.

**Table 1:** Empirical testability of the propositions.

Proposition	Interview-based indicators	Qualitative examination strategy	Supporting and challenging evidence
P1. Lower psychological safety is associated with lower recognition capacity.	Perceived speaking-up risk and futility; retaliation concerns; use of voice and reporting channels; whether signals reach organizational decision-makers.	Compare organizational cases or units with higher and lower psychological safety and reconstruct the sequence from perceived risk to withholding and weak recognition.	Support: Low safety co-occurs with withholding, fragmented information, and weak recognition. Challenge: Recognition remains reliable despite low safety, or remains weak despite high safety for other reasons.
P2. Organizational silence links low psychological safety to low recognition capacity.	Privately shared knowledge; collective expectations of silence; informal discussion versus formal escalation; aggregation of local experiences into organizational knowledge.	Use cross-level process tracing to examine the sequence: low psychological safety → shared silence → failure to create organizational knowledge.	Support: Shared silence interrupts escalation, validation, or aggregation. Challenge: Knowledge reaches the organization despite silence, or recognition failure is better explained by ambiguity or weak diagnostics.
P3. Institutional decoupling weakens intervention capacity.	Formal policies and complaint channels; actual investigations, decision rights, sanctions, follow-up, and perceived credibility of accountability structures.	Compare formal procedures with interview reconstructions of recognized cases from complaint through investigation, decision, sanction, and follow-up.	Support: Formal structures are symbolic or inconsequential and recognized cases do not trigger credible action. Challenge: Formal structures consistently produce intervention, or failure occurs without decoupling.
P4. Useful illegality reduces intervention capacity when organizations depend on informal deviations.	Tolerated rule deviations; operational workarounds; perceived necessity; leader indispensability; anticipated disruption or exposure if intervention occurs.	Trace whether dependence on informal deviations influences decisions not to investigate, sanction, remove, or restrict a destructive leader.	Support: Leaders are protected because they sustain or conceal necessary gray-zone practices. Challenge: Useful illegality is unrelated to intervention, or leaders are sanctioned despite such dependence.
P5. Normalization of deviance reduces recognition and intervention capacity.	Changing tolerance thresholds; rationalization; socialization; conduct becoming described as normal or necessary; changes in reporting and sanctioning over time.	Use retrospective event reconstruction and compare longer-tenured and newer members to identify movement from initial concern to routine acceptance.	Support: Repetition produces desensitization, declining reporting, and reduced sanctioning. Challenge: Harm remains visible and sanctionable despite repetition, or normalization does not affect response capacities.
P6. Resource scarcity and hierarchical pressure increase the likelihood of paralyzed responses in healthcare.	Staffing and resource scarcity; hierarchy; awareness of harm; leader indispensability; short-term stability; delayed or avoided intervention.	Compare healthcare cases or units with different levels of scarcity and hierarchical pressure and examine whether recognized harm is tolerated for operational continuity.	Support: High pressure coincides with acknowledged harm, perceived indispensability, and postponed intervention. Challenge: Intervention occurs despite high pressure, or paralysis is more common in lower-pressure cases.
P7. Persistent blindness and paralysis can move organizations toward incapability through feedback loops.	Repeated unresolved incidents; changes in trust and voice; spread of informal exceptions; diffusion of destructive practices; weakening governance over time.	Apply retrospective or longitudinal within-case process tracing and cross-case comparison to reconstruct temporal ordering and feedback mechanisms.	Support: Repeated non-response is followed by declining trust and voice, normalization, and weakening of both capacities. Challenge: Types remain stable or improve, or deterioration is better explained by external shocks.

P = proposition. The indicators and test strategies are theoretically derived and are intended to guide future empirical examination through semi-structured qualitative interviews. Relational propositions require comparative case analysis, whereas dynamic propositions require retrospective or longitudinal process reconstruction. Supporting evidence should be assessed alongside negative cases and plausible rival explanations.

Healthcare is used as a critical organizational context and theoretical magnifier, not as empirical proof of the framework. Professional hierarchies shape who can speak, what can be said, and how concerns travel, while regulatory complexity, resource constraints, ethical obligations, and operational interdependence intensify the tension between formal standards and continuity of care (Essex et al., 2023; Lainidi et al., 2023; Hague et al., 2025). Recent reviews and studies associate destructive or toxic leadership with burnout, workforce instability, nurse retention, care quality, and patient safety, although evidence for organizational and patient outcomes remains less developed than evidence for staff outcomes (Hult et al., 2023; Labrague, 2023; Palvimo et al., 2023; Tsapnidou et al., 2025). Healthcare makes the proposed recognition and intervention mechanisms especially visible without limiting the framework to this sector.

## 3 Defining organizational inaction

Organizational inaction is defined here as the systematic failure of an organization to recognize, process, escalate, or intervene against destructive leadership despite the availability of signals, experiences, or structural indicators that harmful leadership is occurring. The construct contains both a perceptual component and an action component. The perceptual component concerns whether harmful leadership is interpreted as destructive leadership or as toughness, pressure, or a personality conflict. The action component concerns whether this recognition is translated into credible, timely, and consequential organizational intervention.

The construct has two dimensions. Recognition capacity refers to the organization's capacity to collect, interpret, and validate signals of destructive leadership. It depends on voice channels, psychological safety, leadership diagnostics, sense-making routines, and the credibility of feedback systems. Intervention capacity refers to the organization's capacity to transform recognition into organizational action. It depends on authority, governance, sanctioning mechanisms, independence of control bodies, escalation procedures, and the willingness to accept short-term disruption in order to prevent longer-term damage.

Organizational inaction is related to, but not identical with, organizational silence. Organizational silence describes the collective withholding of information by employees about potential problems or issues (Morrison and Milliken, 2000). More recent research confirms that voice and silence are related but conceptually distinct responses whose occurrence depends on both individual assessments and organizational conditions (Morrison, 2023). It is primarily a communication and voice phenomenon. Organizational inaction is broader. It includes silence as one mechanism that can reduce recognition capacity, but it also includes situations in which information is already available and still no intervention follows. In other words, organizational silence explains why signals may not travel upward, whereas organizational inaction explains why signals may either not be recognized or not be converted into action.

The construct also differs from institutional inertia and the organizational bystander effect. Institutional inertia emphasizes persistence, path dependency, and resistance to change (Hannan and Freeman, 1984), whereas organizational inaction in the present argument is specifically tied to destructive leadership and to the two capacities of recognition and intervention. The organizational bystander effect emphasizes the diffusion of responsibility among multiple actors confronted with problematic situations (Darley and Latané, 1968). In contrast, the concept of organizational inaction proposed here shifts the analytical focus from individual responsibility diffusion to organizational processes of recognition, escalation, and intervention. Organizational inaction is not simply passivity. It is a patterned failure of perception, interpretation, escalation, and accountability.

These boundaries are important for the theoretical contribution. A theory of destructive leadership needs to explain not only why harmful leaders emerge or why employees may remain silent, but also why organizations fail to process available signals and why formal control structures do not always produce effective intervention.

## 4 Destructive leadership and the expansion of leadership research

Destructive leadership is no longer understood as an exceptional deviation at the margins of leadership research. It has become increasingly clear that harmful leadership behavior takes multiple forms and cannot be reduced to isolated incidents of incivility. A leader may humiliate employees, manipulate information, create fear, encourage rivalry, claim others' achievements, withhold recognition, or use pressure and intimidation as routine instruments of influence. Such behavior may be open and aggressive, but it may also be indirect, strategic, and difficult to observe from outside. What unites these forms is that they systematically damage employees, organizations, or both (Spengler et al., 2019; Schilling and Schyns, 2021).

The definitional literature reflects this complexity. Einarsen et al. (2007) define destructive leadership as repeated and systematic leader behavior that undermines the legitimate interests of the organization or the wellbeing and motivation of followers. Especially in the scientific literature about the healthcare sector such leadership forms are defined as harmful behaviors, that damage staff wellbeing and organizational functioning as well as care quality and patient safety (Tsapnidou et al., 2025; Labrague, 2023).

May et al. (2016) emphasize the processual nature of the phenomenon and stress that destructive leadership is experienced by employees as obstructive or hostile. Externbrink (2019) has added an important clarification by arguing that destructive leadership may also consist in systematic omission, that is, in the sustained failure to fulfill leadership responsibilities where such failure causes damage to employees and organizational structures. This broader perspective is particularly relevant because harmful leadership is not always noisy. Sometimes the destructive element lies in neglect, indifference, or strategic non-intervention.

The field is also characterized by a proliferation of neighboring terms. Abusive supervision, petty tyranny, toxic leadership, strategic bullying, pseudotransformational leadership, and managerial tyranny each highlight a somewhat different aspect of the same broader phenomenon. Abusive supervision focuses on sustained hostile verbal and non-verbal behavior. Petty tyranny emphasizes arbitrariness and humiliation. Toxic leadership draws attention to manipulation, dependence, and the broader contamination of the organizational environment. Strategic bullying points to the instrumental use of aggression. Despite these differences, the overlap between the constructs is substantial. This overlap supports the use of destructive leadership as an umbrella term (Tepper, 2000; Ashforth, 1994; Ma et al., 2004; Thoroughgood et al., 2018).

An important implication of this conceptual diversity is that destructive leadership cannot be captured by a single behavioral indicator. Employees may perceive highly similar acts differently depending on context, and some leader behaviors may appear functional in the short term while proving destructive in retrospect. Particularly in high-pressure fields, leaders who produce short-term results through fear, information control, or coercion may initially be evaluated positively by superiors even while employees already experience the situation as harmful. Such ambiguity is one reason why organizations struggle not only to intervene, but even to identify destructive leadership in the first place (May et al., 2016; Nerdinger, 2014a).

Another implication concerns the level of analysis. Early discussions often drifted toward person-centered explanations, as if destructive leadership were reducible to the pathology of individual leaders. More recent work, however, has criticized this tendency and called for broader frameworks (Padilla et al., 2007). Thoroughgood et al. (2018), in particular, have argued that leader-centric approaches underestimate the roles of followers, organizational conditions, and contextual constraints. This critique is crucial for the argument developed in the present paper. If destructive leadership is not a purely individual phenomenon, then the organizational failure to address it must also be part of the explanation.

## 5 Explanatory traditions: leader, follower, environment

The current research field offers at least three major explanatory traditions: leader-centered explanations, follower-centered explanations, and environment-centered explanations (Padilla et al., 2007). Each of these traditions captures a relevant part of the problem, but none of them sufficiently theorizes organizational inaction (Helfrich and Steidle, 2017).

Leader-centered explanations are most prominently represented by the literature on the dark triad. Narcissism, Machiavellianism, and psychopathy are treated as three personality dimensions that, in certain combinations or intensities, may predispose leaders toward harmful forms of influence. Narcissistic leaders are characterized by an unstable but inflated self-image, a need for admiration, and hostile reactions to criticism. Machiavellian leaders are strategic, cynical, and willing to manipulate others to achieve their own goals. Psychopathic leaders, especially in their subclinical or corporate forms, display emotional coldness, fearlessness, low guilt, and a readiness to violate rules. Research has repeatedly suggested that positions of power can attract such personalities and that organizational selection systems often fail to screen them adequately (Externbrink and Keil, 2018; Spengler et al., 2019).

Yet destructive personality traits do not translate automatically into destructive organizational outcomes. Their effects are mediated by opportunities, norms, and dependencies. This leads to follower-centered explanations. The literature on susceptible followers suggests that destructive leaders rely on different types of support or compliance. Some followers actively collaborate because they expect personal benefit, prestige, or protection. Others conform more passively out of fear, dependency, insecurity, or the desire to preserve their place within the group (Jonason et al., 2014). Padilla et al. (2007) distinguish between colluders and conformers to capture this difference. The distinction is important because it shows that destructive leadership is not sustained solely by fear; it may also be reinforced by ambition, identification, or strategic alignment.

Follower susceptibility is not a static individual characteristic. It is often produced and reinforced through organizational processes. Learning effects are particularly relevant here. If leaders themselves have experienced destructive supervision, a trickle-down effect may develop in which such behavior is reproduced at lower hierarchical levels. Likewise, if employees repeatedly observe that pressure, intimidation, or manipulation are rewarded, those practices may become normalized. The result is not just behavioral imitation but a shift in organizational expectations: what was initially perceived as deviant can become the accepted way of managing (Bandura, 1986; May et al., 2016).

Environment-centered explanations complete the picture. The toxic triangle brings all three components together by describing destructive leadership as the result of destructive leaders, susceptible followers, and conducive environments. The environmental side of the triangle includes instability, perceived threat, cultural values, and the absence of effective controls. These factors do not create destructive leadership by themselves, but they lower the barriers against it. Under conditions of crisis, uncertainty, and hierarchical concentration of power, authoritarian or manipulative leadership may gain legitimacy. Likewise, where control systems are weak or symbolic, destructive leaders can remain unchallenged even when their behavior is widely experienced as harmful (Padilla et al., 2007; Thoroughgood et al., 2018).

The strength of these traditions lies in their cumulative insight: destructive leadership is relational and contextual. Their weakness, however, is equally clear. They explain why harmful leaders can emerge and sometimes why followers do not resist them, but they say comparatively little about why organizations as systems fail to process the problem. The organization often appears merely as the environment in which destructive leadership happens. The present paper argues that this is insufficient. Organizations must be analyzed not only as contexts, but as actors with capacities, blind spots, and defensive routines of their own (Thoroughgood et al., 2018; Tayfur Ekmekci and Güney, 2021).

## 6 Organizational silence and psychological safety as recognition mechanisms

Morrison and Milliken (2000) define organizational silence as a collective phenomenon in which employees withhold information about potential organizational problems. Their model is directly relevant because destructive leadership can remain unrecognized when employees consider speaking up futile or dangerous. Later work on employee silence similarly shows that employees often do not communicate upward about important issues because they fear negative consequences, doubt that change will occur, or perceive that managers do not want to hear the information (Milliken et al., 2003).

Organizational silence is a central antecedent of low recognition capacity. If affected employees remain silent, destructive leadership may exist as local experience without becoming organizational knowledge. However, the present model goes beyond organizational silence by adding the intervention dimension. Even where silence is overcome and destructive leadership becomes known, organizations may still fail to act. This is the central extension offered by the concept of organizational inaction: it connects the failure of upward information processing to the failure of governance and intervention.

Psychological safety further specifies the conditions under which recognition capacity can develop. Edmondson (1999) conceptualizes psychological safety as a shared belief that the team is safe for interpersonal risk taking. In the context of destructive leadership, psychological safety is relevant because recognition depends on whether employees, peers, and subordinate managers can raise concerns without expecting retaliation, ridicule, or career damage. Detert and Edmondson (2011) show that employees may hold implicit voice theories, that is, taken-for-granted beliefs about why speaking up is inappropriate or risky. Such beliefs can become particularly powerful in hierarchical organizations.

Recognition capacity rests on a chain of mechanisms. Employees must perceive harmful conduct, interpret it as organizationally relevant, believe that speaking up is legitimate, trust that channels are safe, and expect that the organization can respond. Weak psychological safety interrupts this chain before recognition can become organizational. Low psychological safety thus does not merely create discomfort; it lowers the probability that destructive leadership becomes visible to the system.

## 7 Dark triad, toxic process, and organizational gravity

The literature on the dark triad has been especially influential because it gives concrete form to the notion of destructive leader dispositions. At first sight, the attraction of this literature is obvious. It names the leader characteristics that many practitioners intuitively associate with harmful forms of authority: manipulation, excessive self-focus, emotional coldness, and strategic exploitation. Yet the literature also contains a warning. It repeatedly shows that these traits can be misread as strengths under the conditions of organizational competition (Externbrink and Keil, 2018; Helfrich and Steidle, 2017).

This paradox appears most clearly in the case of narcissism. Narcissistic leaders often present themselves confidently, speak in visionary terms, and perform well in impression-management situations. Especially in unstructured interviews or poorly designed promotion procedures, such self-presentation can be confused with leadership potential. Machiavellian leaders likewise benefit from their strategic realism, their ability to calculate advantage, and their lack of scruples. Corporate psychopaths may appear calm under pressure, socially potent, and unusually fearless. In settings that reward decisiveness and emotional distance, these features can be perceived positively, at least in the beginning (Spengler et al., 2019; Boddy, 2021).

Schüler-Lubienetzki and Lubienetzki (2017) describe the development of destructive leadership as a toxic process with several phases. It begins with exploration and the identification of vulnerabilities in the surrounding system. It then proceeds to manipulation, in which loyal supporters are cultivated, critics are isolated, and fear becomes a governing medium. In a later phase, the destructive leader consolidates power and begins to harvest the advantages of the position. Only in the final phase does the structure of deception collapse under the weight of damage, conflict, and organizational dysfunction. This model is useful because it emphasizes temporal dynamics. Destructive leadership does not always appear fully formed. It may begin under the cover of competence and only later reveal its full implications.

The same temporal logic appears in research on performance evaluations. Leaders with destructive traits may initially receive highly positive assessments because evaluators anchor their judgments in first impressions and because formal outcomes seem favorable. Only later, when turnover rises, trust declines, and conflict intensifies, does the destructive character of the leadership style become more apparent. This delay is crucial for the theory of organizational inaction developed here. If destructive leadership is often recognized only retrospectively, then blind organizations are not an empirical anomaly. They are a predictable outcome of evaluation systems that privilege appearance, short-term output, and top-down impression management (Spengler et al., 2019; Bierhoff, 2007).

A further concept that deserves more attention is organizational gravity (Nerdinger, 2014b). Drawing on the attraction-selection-attrition model, this concept captures how organizations reproduce certain personality structures and behavioral norms. Leaders tend to attract, select, and promote individuals who fit existing expectations and increase the internal coherence of the system. This mechanism is not neutral. If destructive tendencies already exist in positions of authority, the organization may gradually become more hospitable to similar actors. Those who do not fit the prevailing pattern either fail to enter, fail to rise, or leave. Over time, the organization becomes gravitationally aligned with a certain leadership culture (Schneider et al., 2000).

Organizational gravity matters because it links individual dispositions to organizational reproduction. It explains why destructive leadership can become concentrated in specific settings without presupposing that all actors are equally destructive. Once destructive actors shape recruitment, promotion, and informal norms, they can alter the probability distribution of future leadership appointments. In this sense, destructive leadership may become self-reinforcing. The problem is no longer a single leader, but a cumulative organizational tendency (Schneider et al., 2000; Externbrink and Keil, 2018).

Organizational gravity is treated as a mechanism that influences both recognition and intervention. It affects recognition because selection and promotion routines shape which behaviors are interpreted as normal. It affects intervention because leaders selected into a similar behavioral culture may have reduced motivation to sanction conduct that resembles their own implicit norms. Organizational gravity therefore changes the probability distribution of organizational types over time: repeated selection of similar leadership profiles makes blindness and paralysis more likely.

## 8 Environmental conditions, culture, and control

The environmental side of destructive leadership research points to the fact that organizations differ in the extent to which they facilitate harmful leadership. Crisis, perceived threat, hierarchy, cultural expectations, and weak control mechanisms do not mechanically produce destructive leadership, but they lower the threshold for its emergence and persistence (Padilla et al., 2007; van Rooij and Fine, 2018).

Perceived threat is especially important. Threat does not have to be objectively real in order to have organizational effects. It may be communicated, symbolically amplified, or selectively interpreted. In organizations under economic pressure, facing restructuring, staff shortages, or external criticism, leaders may justify authoritarian behavior as necessary for survival. In such situations, followers may accept pressure and fear-based leadership because they believe the organization is in danger. This turns threat into a legitimizing device. It also opens the door for leaders to convert uncertainty into power (Padilla et al., 2007; Thoroughgood et al., 2018).

Closely related is the role of injustice and displaced aggression. Leaders who themselves experience unfair treatment by higher levels may redirect their frustration downward. Instead of confronting the actual source of the problem, they channel aggression toward subordinates who are less able to defend themselves. This mechanism can produce a trickle-down dynamic in which destructive behavior cascades through hierarchical levels. Such processes matter for organizational inaction because they demonstrate that destructive leadership may be rooted in authority structures as well as in individual personality characteristics (Nerdinger, 2014a; Schilling and Schyns, 2021).

Cultural values provide another layer of explanation. Research inspired by Hofstede (2001) and by the GLOBE project (House et al., 2004) suggests that high power distance, strong uncertainty avoidance, and certain forms of collectivism may increase tolerance toward destructive leadership. Where asymmetrical power relations are broadly accepted, criticism of leaders becomes less likely (Hofstede, 2001). Where uncertainty avoidance is high, strong leadership may be preferred even when it takes harsh forms (House et al., 2004). Cultural patterns do not determine behavior directly, but they shape what is perceived as legitimate, effective, or normal. In this way, they can reduce recognition and resistance.

Control mechanisms represent the final element in this cluster. Internal controls include boards, supervisory arrangements, ethical guidelines, leadership reviews, and fraud prevention systems (Schmid and Pircher Verdorfer, 2020). External controls include media scrutiny, regulators, and independent experts. In theory, such controls should limit destructive leadership. In practice, they often fail (Appelbaum and Roy-Girard, 2007). Control bodies may be dependent, underinformed, politically constrained, or absent at the very levels where concentration of power is highest. Senior leaders in particular may operate in spaces with little effective oversight. Under such conditions, destructive leadership is not only more likely to arise; it is more difficult to interrupt.

The distinction between formal and effective controls is crucial. Many organizations possess formal structures that signal control without actually generating accountability (Spengler et al., 2019). Employee surveys may exist but not lead to consequences. Leadership principles may be formulated but remain too vague to guide intervention. Complaint channels may be available but not trusted. In terms of the model developed in this paper, such conditions are likely to produce paralysis instead of functionality. The organization sees the problem, but the formal architecture is too weak, too compromised, or too symbolic to transform recognition into action.

Yet, formal control structures do not automatically create intervention capacity. They create intervention capacity only when they are coupled with authority, independence, information access, and consequences. Where formal structures exist mainly as symbols of legitimacy, they may create the appearance of control while leaving destructive leadership practically untouched.

## 9 Institutional decoupling and normalization of deviance

Institutional theory clarifies why organizations may maintain formal structures that signal compliance without producing effective accountability. Meyer and Rowan (1977) argue that formal structures can function as myths and ceremonies that secure legitimacy in the institutional environment. DiMaggio and Powell (1983) further show that organizations may adopt similar structures through coercive, mimetic, and normative pressures. Applied to destructive leadership, this means that leadership principles, ethics codes, employee surveys, complaint channels, and governance committees may exist because they are institutionally expected, not because they reliably transform harmful leadership into corrective action.

This distinction between formal structures and effective intervention is central to the present theory. A healthcare organization may possess a mission statement, compliance system, leadership competency model, or whistleblowing channel and still remain paralyzed if these structures are not trusted, not independent, or not consequential. Institutional decoupling therefore explains why organizations may appear functional from the outside while remaining unable to act internally. It also explains why the existence of a formal intervention architecture should not be confused with intervention capacity.

The literature on normalization of deviance and organizational misconduct deepens this mechanism. Vaughan (1999) shows that routine non-conformity can become embedded in organizations and produce harmful outcomes through ordinary organizational processes. Anand et al. (2004) describe how deviant practices can become accepted and perpetuated as business as usual through rationalization, socialization, and institutionalization. These mechanisms are relevant because destructive leadership often persists not as a dramatic violation, but as a gradually normalized pattern of pressure, humiliation, fear, and rule bending.

Normalization of deviance links recognition failure and intervention failure. It reduces recognition because harmful practices are reinterpreted as normal, necessary, or effective. It reduces intervention because deviant practices become embedded in routines, dependencies, and informal expectations. In this way, institutional decoupling and normalization of deviance provide the organizational-theoretical foundation for the paper's central claim: organizations do not merely fail to respond to destructive leadership; they may create the conditions under which such leadership becomes difficult to see and costly to stop.

## 10 Useful illegality as a missing mechanism

One of the most original and analytically promising concepts for understanding organizational inaction is useful illegality. The concept, associated with systems-theoretical thinking, refers to deviations from formal rules that are tolerated because they appear to stabilize organizational functioning (Luhmann, 2022; Kühl, 2019). Organizations are never governed by rules alone. Their formal order is constantly complemented, bypassed, or corrected by informal practices that make work possible under conditions of complexity and contradiction (Kühl, 2019). In many cases, such deviations are not anomalies but normal features of organizational life.

The problem begins when such gray zones become normalized and when the line between compensatory flexibility and harmful conduct becomes blurred. Useful illegality affects destructive leadership in three related ways. Widespread rule bending can obscure the boundary between pragmatic deviation and coercive conduct, allowing destructive leaders to hide within a broader culture of informal exception (Straub and Lemmer, 2016). Organizational dependence on these deviations can also discourage intervention, since decisive action would expose practices on which daily operations rely. Over time, tolerated deviations may change the moral coding of harmful behavior, making conduct appear necessary or even functional instead of unacceptable. From this perspective, useful illegality is not simply a side issue. It is a mechanism through which organizations become implicated in the conduct they later fail to sanction. This complicity need not be conscious or cynical. It may be systemic (Luhmann, 2022).

Useful illegality is specified as a mechanism that primarily weakens intervention capacity, while indirectly weakening recognition capacity. It weakens intervention because the organization may depend on the same informal deviations that would need to be exposed if destructive leadership were sanctioned. It weakens recognition indirectly because repeated tolerance of gray-zone conducts changes what members perceive as normal or necessary. Useful illegality therefore links informal functionality to formal non-response.

## 11 Healthcare as a critical case and theoretical magnifier

The healthcare sector is not used here as empirical proof for the proposed theory. It is used as a critical case and theoretical magnifier. Healthcare combines strong professional ethics with persistent resource scarcity, making tensions between formal norms and practical necessities particularly visible. This means that healthcare makes the mechanisms of organizational inaction especially visible because it combines hierarchy, moral pressure, interdependence, resource scarcity, regulation, and informal workarounds in a concentrated form.

Healthcare organizations must reconcile economic pressure with professional ethics. They are expected to use resources efficiently while simultaneously ensuring safety, dignity, continuity, and quality of care. These goals are not always easy to align. Leaders who promise to secure short-term results under difficult conditions may receive legitimacy even when they use harmful means (Frodl, 2013; Herzka, 2013). Care delivery also depends on a high degree of interdependence. Care processes depend on coordination between professional groups, between shifts, and between formal and informal routines. Yet the same style often damages precisely the trust and psychological safety that complex coordination requires. The contradiction is central: what appears efficient in the short term may be destructive in the long term (Bär and Starystach, 2017; Holloway and Kusy, 2010). Pronounced hierarchical structures add another source of organizational vulnerability. Medical authority, nursing management, administrative governance, and technical support functions often intersect in ways that produce asymmetrical communication structures. Employees at lower levels may experience destructive conduct clearly while lacking secure channels to communicate it upward. This can generate blindness at higher levels and resignation at lower ones. Where leadership is strongly personalized and role expectations are steep, upward challenge becomes risky (Frodl, 2013; Holloway and Kusy, 2010). Healthcare work is emotionally and morally demanding. This professional commitment can unintentionally support organizational silence when employees avoid reporting destructive leadership because they fear burdening colleagues or destabilizing already fragile staffing situations (Holloway and Kusy, 2010; Frodl, 2013; Palvimo et al., 2023). Finally, healthcare is structurally prone to useful illegality. Staff shortages, emergencies, documentation demands, and contradictory regulatory expectations often require local improvisation. Such improvisation may preserve care continuity, but it can also create gray zones in which formal controls and escalation rules become less stable (Luhmann, 2022; Kühl, 2019; Straub and Lemmer, 2016).

These sector-specific conditions do not imply that destructive leadership is unique to healthcare. Rather, healthcare functions as a theoretical magnifier: it makes visible how recognition deficits, intervention deficits, and useful illegality can combine in the organizational processing of destructive leadership.

## 12 A typological model of organizational inaction

Research on organizational silence (Morrison and Milliken, 2000), psychological safety (Edmondson, 1999), institutional decoupling (Meyer and Rowan, 1977), normalization processes (Vaughan, 1999; Ashforth and Anand, 2003), and destructive leadership (Padilla et al., 2007) collectively suggests that organizations differ in their capacity to recognize and respond to leadership-related harm.

The resulting model distinguishes organizations along two continuous dimensions: their capacity to recognize destructive leadership and their capacity to intervene against it. The four organizational configurations—functional, blind, paralyzed, and incapable organizations—are presented as ideal-typical constructs intended to organize and extend current knowledge on organizational responses to destructive leadership. Their purpose is to provide a theoretically grounded framework that can guide future empirical investigation across healthcare and other organizational contexts.

Recognition capacity is grounded in research on organizational silence and psychological safety. Morrison and Milliken (2000) demonstrated that organizations frequently fail to process upward information about problems because structural and cultural conditions discourage voice behavior. Similarly, Edmondson (1999) showed that psychological safety strongly influences whether employees communicate concerns, admit mistakes, and challenge problematic conduct. Subsequent meta-analytic evidence has confirmed that psychological safety is positively associated with voice behavior, learning, information sharing, and error reporting across a wide range of organizational settings (Frazier et al., 2017). Together, these perspectives suggest that organizational failures to recognize destructive leadership are not simply individual perceptual errors but emerge from broader organizational conditions that shape information processing.

Intervention capacity is grounded in research on organizational governance, institutional accountability, and the enforcement of organizational norms. Institutional theory suggests that organizations frequently decouple formal structures from actual practice, creating situations in which recognized problems do not automatically trigger corrective action (Meyer and Rowan, 1977). Research on normalization processes further demonstrates that organizations may tolerate and reproduce harmful practices even when they are widely known, particularly when such practices become embedded in routines and perceived as functionally necessary (Vaughan, 1999; Ashforth and Anand, 2003). From this perspective, intervention capacity reflects the ability of an organization to transform recognition into corrective action. Organizations may recognize destructive leadership while simultaneously lacking the governance structures, accountability mechanisms, or political willingness necessary to respond effectively. Intervention failures are thus not reducible to ignorance; they emerge from organizational processes that prevent action despite awareness.

The four ideal types represent analytically distinct configurations of recognition and intervention capacity. Their purpose is not to classify organizations definitively but to provide a theoretical framework that can guide empirical testing and further theory development (Weber, 1949; Doty and Glick, 1994).

The core argument of this article can now be stated more precisely. Organizations differ systematically along two continuous dimensions: their ability to recognize destructive leadership and their ability to intervene against it. Recognition refers to the capacity to identify destructive leadership as such. Intervention refers to the capacity to translate recognition into effective organizational action. When both dimensions are crossed, four heuristic ideal types emerge. These types should not be understood as empirically discrete categories with fixed thresholds. They are analytical ideal types that clarify patterns of organizational response and provide a basis for empirical testing.

Functional organizations are able both to recognize and to confront destructive leadership. They possess structured and credible procedures for assessing leadership behavior, for identifying warning signs, and for escalating concerns. Their control instances are not merely symbolic. Feedback systems, employee surveys, leadership standards, training measures, and supervisory structures form a coherent architecture. Functional organizations are less likely to rely systematically on useful illegality, because informal workarounds do not systematically undermine formal order. In such contexts, destructive leaders encounter resistance early and find it difficult to stabilize their position (May et al., 2016; Craig and Gustafson, 1998).

Blind organizations are unable to recognize destructive leadership adequately, although they would in principle be able to intervene if the phenomenon were identified correctly. Their main deficit lies in diagnosis and interpretation. The organization may possess functioning structures, control bodies, and even a comparatively well-articulated leadership culture. Yet destructive conduct remains invisible because evaluation standards are too weak, instruments too superficial, or harmful behavior is reframed as strength, toughness, or ordinary managerial pressure. Blind organizations are not structurally helpless, but perceptually deficient (Externbrink and Keil, 2018; Spengler et al., 2019; Morrison and Milliken, 2000).

Paralyzed organizations recognize destructive leadership but fail to act. Their awareness may result from complaints, diagnostic procedures, or broad informal knowledge. Nevertheless, intervention does not take place. This may be due to political dependencies, fear of destabilization, weak sanctioning procedures, missing rules, institutional decoupling, or the absence of legitimate authority to act. Paralyzed organizations are especially important because they show that knowledge alone is insufficient. They also tend to display high levels of useful illegality, since rule deviations may be necessary to keep the organization functioning and intervention would expose this dependency (Bildat and Torka, 2019; Padilla et al., 2007; Meyer and Rowan, 1977; Kühl, 2019).

Incapable organizations combine both deficits. They neither recognize destructive leadership reliably nor possess functioning intervention capacities. Selection procedures are weak, control mechanisms ineffective or absent, leadership culture fragmented, and goal structures unclear. Such organizations often display high turnover, chronic inefficiency, and a broad diffusion of destructive practices across hierarchical levels. The problem is no longer localized in a few leaders; it has become embedded in the system. The incapable organization represents the maximum escalation of organizational inaction (van Rooij and Fine, 2018; Vaughan, 1999).

Figure 1 summarizes the placement of the four ideal types across the recognition and intervention dimensions.

**Figure 1:**
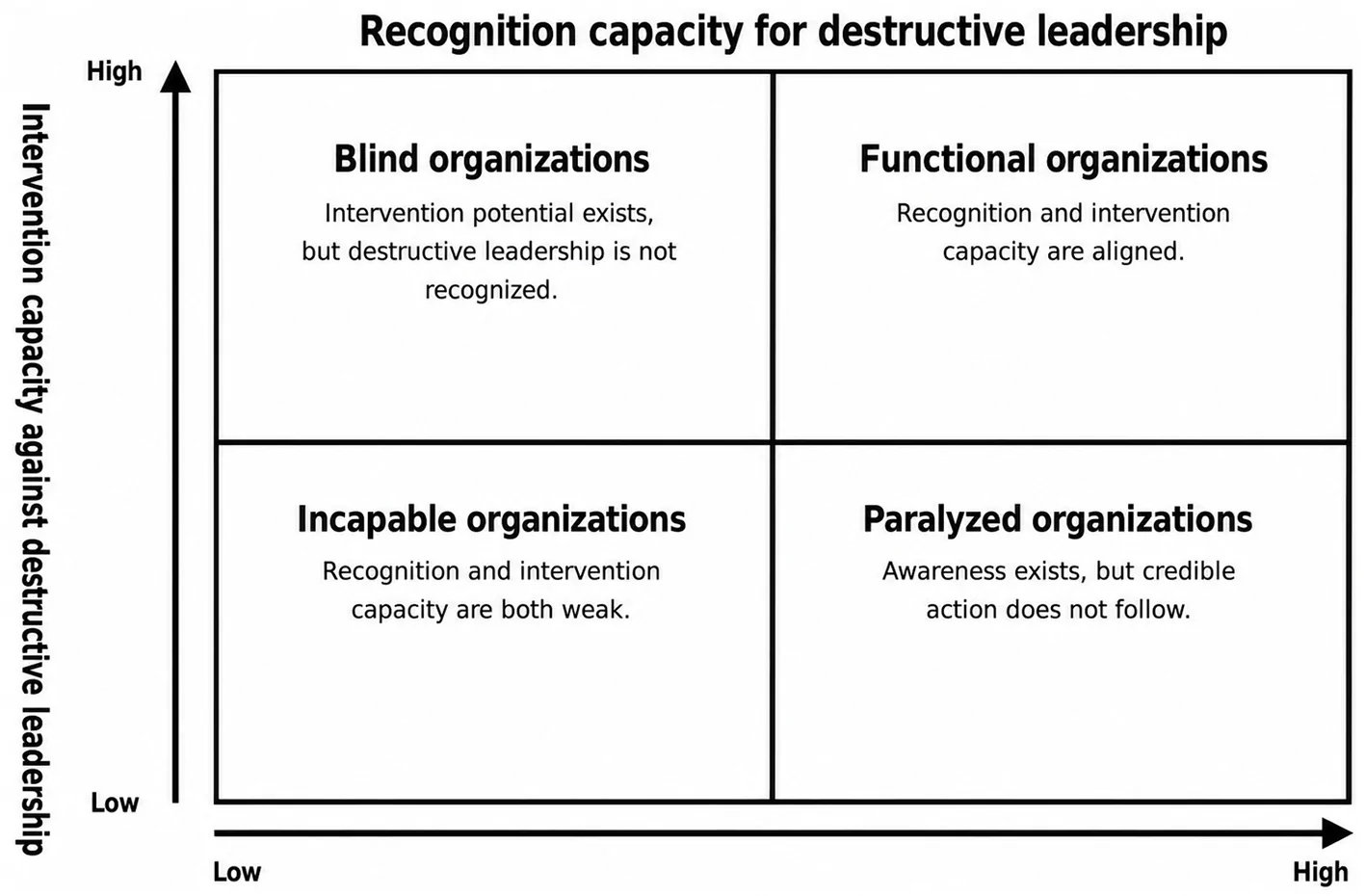
Typological model of organizational inaction in destructive leadership. The model positions four heuristic ideal types on continuous recognition and intervention capacities.

The typology is more dynamic than static. Blind organizations may become functional if recognition improves, for example through better diagnostics, psychological safety, and leadership education. Paralyzed organizations may become functional if they establish credible intervention pathways and independent controls. Conversely, long-term blindness or paralysis may move an organization toward incapability. This developmental logic is one reason why the typology is analytically useful. It not only differentiates existing states, but also provides a framework for understanding organizational trajectories (Vaughan, 1999).

## 13 Formal propositions and dynamic mechanisms

The relationships derived from the preceding theoretical synthesis are summarized in seven propositions. Together, they specify how organizational conditions shape recognition and intervention capacity and provide a basis for subsequent empirical examination.

Proposition 1: Lower psychological safety is associated with lower recognition capacity because employees are less likely to communicate upward about destructive leadership when they perceive speaking up as risky or futile (Edmondson, 1999; Morrison and Milliken, 2000).

Proposition 2: Organizational silence functions as a mechanism linking low psychological safety to low recognition capacity. Where silence becomes collectively shared, destructive leadership is more likely to remain a local experience rather than become organizational knowledge (Milliken et al., 2003; Detert and Edmondson, 2011).

Proposition 3: Institutional decoupling weakens intervention capacity by creating a divergence between formal control structures and effective accountability. Organizations may possess ethics codes, complaint channels, and leadership standards without translating them into credible action (Meyer and Rowan, 1977; DiMaggio and Powell, 1983).

Proposition 4: Useful illegality reduces intervention capacity when organizations become dependent on informal deviations that sustain daily functioning. In such cases, sanctioning destructive leadership would also expose the organization's reliance on gray-zone practices (Luhmann, 2022; Kühl, 2019; Straub and Lemmer, 2016).

Proposition 5: Normalization of deviance reduces both recognition and intervention capacity by transforming initially questionable practices into routine expectations. As harmful conduct becomes interpreted as ordinary or necessary, it becomes less visible and less sanctionable (Vaughan, 1999; Anand et al., 2004).

Proposition 6: Healthcare organizations under high resource scarcity and strong hierarchical pressure are more likely to develop paralyzed responses when destructive leaders are perceived as maintaining short-term operational stability despite long-term harm (Essex et al., 2023; Hague et al., 2025; Palvimo et al., 2023; Frodl, 2013; Bär and Starystach, 2017).

Proposition 7: Organizational types are not stable categories. Persistent blindness and persistent paralysis can produce feedback loops that move organizations toward incapability by weakening trust, reducing voice, and normalizing informal exception over time (Thoroughgood et al., 2018; Vaughan, 1999).

These propositions specify the causal mechanisms connecting the literature reviewed above to the typology. Psychological safety and organizational silence primarily affect recognition capacity. Institutional decoupling and useful illegality primarily affect intervention capacity. Normalization of deviance affects both dimensions. Feedback loops explain movement between types over time.

Although theoretically derived, the propositions are formulated in a manner that allows systematic empirical examination. Table 1 summarizes the interview-based indicators, qualitative test strategies, and evidence that would either support or challenge each proposition. Relational propositions require comparative case analysis, whereas dynamic propositions require retrospective or longitudinal process reconstruction. A more detailed interview-based operationalization, including qualitative examination strategies and evidence that would support or challenge each proposition, is provided in Supplementary Table 1.

## 14 Consequences, countermeasures, and the problem of short-term success

Destructive leadership also has consequences that justify why organizational inaction matters beyond the immediate relationship between leader and follower. On the employee side, the literature points to declining motivation, higher strain, emotional exhaustion, fear, withdrawal, silence, and deviant work behavior. Employees under destructive leaders may avoid communication, reduce commitment, and disengage psychologically long before they leave formally. This is important because the damage is often not immediately visible in organizational metrics. Teams may continue to function for some time while trust erodes beneath the surface (Schmid and Pircher Verdorfer, 2020; Paltu and Brouwers, 2020).

On the organizational side, the costs can be substantial. High turnover, absenteeism, conflict, reduced innovation, and deteriorating coordination all affect the capacity of the organization to pursue its goals. Where destructive leadership spreads through imitation or trickle-down dynamics, the impact becomes cumulative and not isolated. In such cases, organizations may gradually lose the very capabilities they need in order to correct the problem. This is another reason why the recognition–intervention distinction matters. The longer destructive leadership remains unchallenged, the more it may impair the organization's own ability to perceive and act (Appelbaum and Roy-Girard, 2007; Henriques et al., 2019).

The literature also contains a wide range of countermeasures. At the organizational level, these include clearer leadership principles, robust mission statements, better governance, employee surveys, complaint systems, performance review processes, and more structured diagnostics in recruitment and promotion. At the personnel level, interventions may include coaching, training, reflection on leadership behavior, and stronger support for affected employees. However, the existence of such measures does not guarantee implementation. The barriers to implementation are themselves theoretically significant. They include cost concerns, weak leadership culture, the absence of credible control bodies, overly vague organizational guidelines, and the fear of losing high-performing but harmful managers (May et al., 2016; Ramos, 2006; Externbrink and Keil, 2018).

These barriers indicate that organizational inaction does not primarily reflect a lack of available countermeasures. It emerges when organizations lack the structural and cultural conditions required to make such measures credible, legitimate, and effective. This is precisely what differentiates a merely technical problem from a structural one. The challenge is not only to identify better interventions, but to understand why existing interventions often remain unused (Vennemann and Brouns, 2019; Bildat and Torka, 2019).

Another issue that deserves mention is the distinction between short-term organizational success and long-term organizational health. Several authors have noted that destructive leadership may in some cases appear effective in the short run. Fear can increase compliance, coercion can accelerate decisions, and manipulation can produce visible results. From the perspective of top management, this may create a dangerous illusion. The leader appears strong, decisive, and goal oriented, while the long-term damage remains diffuse or is attributed to external conditions. Such dynamics are particularly relevant in organizations that prioritize immediate target attainment over cultural sustainability (Externbrink, 2019; Boddy, 2021).

This short-termism also helps explain why destructive leadership may occasionally be desired. Organizations are unlikely to openly embrace destructiveness as a principle. Under specific conditions, organizations may tolerate or even reward leadership behavior that only produces short-term benefits. In this respect, the theory intersects with the notion of useful illegality. Both concepts point to a divergence between formal values and practical tolerance under pressure (Straub and Lemmer, 2016; Kühl, 2019).

At the same time, the literature warns against confusing temporary compliance with genuine organizational effectiveness. Where fear, manipulation, and rule bending become regular instruments of leadership, the apparent gains are usually purchased at the cost of trust, learning, innovation, and retention. The organization may still achieve isolated targets, but it becomes less resilient, less reflective, and more dependent on informal pressure. What appears as efficiency may therefore be a hidden form of organizational depletion (Appelbaum and Roy-Girard, 2007; Henriques et al., 2019).

The typology developed in this article can be used to reframe this tension. Blind organizations may misread short-term results as evidence that nothing is wrong. Paralyzed organizations may know the long-term costs but still postpone intervention because the short-term dependency on the destructive leader is too great. Incapable organizations may no longer be able to distinguish performance from damage because the entire organizational order has been reshaped by destructive dynamics. Functional organizations, by contrast, are more likely to resist the seduction of short-term gains because they possess both the instruments and the culture to evaluate leadership in broader terms.

## 15 Discussion

Persistent organizational tolerance of destructive leadership can be understood as a failure of recognition, intervention, or both. Combining these capacities yields four heuristic ideal types—functional, blind, paralyzed, and incapable organizations. The typology shifts attention from the presence of destructive leadership alone to the organizational processes through which harmful conduct becomes visible, actionable, and sanctionable. It complements leader-, follower-, and environment-centered explanations by adding the organization's response capacity to the analysis (Padilla et al., 2007).

Recent outcome research confirms that destructive leadership has substantial effects, but it also shows the limits of an outcome-centered account. In a meta-analysis of 53 studies, Montano et al. (2023) identified destructive leadership as one of the strongest predictors of followers' overall and negative mental health. In a survey of 2,115 Finnish nurses, Palvimo et al. (2023) found positive associations of destructive leadership and job demands with burnout; job resources reduced the strength of both associations. Reviews in nursing also connect toxic leadership with retention problems and patient-safety concerns (Labrague, 2023; Tsapnidou et al., 2025). Hult et al. (2023), however, found that nursing-leadership research still focuses mainly on staff outcomes, with organizational and patient outcomes receiving less attention. These studies establish the consequences of harmful leadership, but they generally begin with exposure and end with outcomes. The present framework examines the intervening organizational process: how warning signals are recognized, combined across levels, and converted—or not converted—into action.

Existing destructive leadership research remains divided across neighboring concepts and explanatory traditions. Leader-centered approaches explain how harmful actors rise and behave, follower-centered approaches explain compliance and support, and environment-centered approaches identify conditions that facilitate destructive leadership. Holistic models such as the toxic triangle bring these domains together, but they are more directly concerned with the emergence and enabling conditions of destructive leadership than with the subsequent organizational processing of available warning signals. Organizational inaction addresses this gap by treating the organization not merely as a context, but as an actor with information-processing, governance, and accountability capacities of its own (Padilla et al., 2007; Thoroughgood et al., 2018; Schilling and Schyns, 2021).

Research on voice and silence is closest to the recognition side of the model, although its findings are not fully uniform. Lainidi et al. (2023) identified heterogeneous concepts and measures across 76 healthcare studies, no unifying theoretical foundation, and limited evidence connecting research with practice. Li et al. (2024) reviewed six studies of negative leadership and silence among nurses or healthcare staff. Four reported positive correlations, whereas one reported a negative correlation; the authors also judged the available evidence to be limited and low in quality. These findings support silence as one route through which destructive leadership can remain locally contained, but they caution against treating it as a single or sufficient explanation. The recognition–intervention distinction accommodates both situations: concerns may fail to become organizational knowledge, or they may be known without producing a credible response.

Evidence on normalization and decoupling helps explain the second situation. Wright et al. (2022) found that productivity pressure, complacency, social pressure, and negative acculturation contributed to normalized deviations in operating rooms, while nurse engagement and supportive managerial relationships were protective. This pattern is consistent with the proposed feedback process in which repeated non-response changes what employees regard as normal. The study did not examine destructive leadership or organizational intervention directly, so the correspondence remains a theoretical extension. Mor Barak et al. (2022), working in inclusion research, model how espoused policies and enacted supervisory practices may diverge. Applied here, that distinction clarifies why formal reporting systems can coexist with weak intervention. The present framework connects these mechanisms by locating normalization across recognition and intervention, and policy–practice decoupling primarily within intervention capacity.

The distinction between blindness and paralysis is particularly important because apparently similar patterns of non-response may arise from different causal configurations. Blindness primarily reflects failures of perception, interpretation, and upward voice, whereas paralysis reflects failures of authority, accountability, or willingness to accept the short-term costs of intervention. Nevertheless, neither type is exclusively psychological or structural. Recognition is influenced by organizational channels and power relations, while intervention is shaped by fear, loyalty conflicts, anticipated career consequences, and institutional constraints. The typology therefore avoids conflating a lack of awareness with an inability or unwillingness to act (Einarsen et al., 2007; Morrison and Milliken, 2000; Weick, 1995; Maitlis and Christianson, 2014).

Healthcare evidence sharpens the model but does not establish it. Essex et al. (2023) show how hierarchy affects who can speak, what can be said, and how concerns are negotiated, while Hague et al. (2025) identify organizational, leadership, team, and individual influences on employee voice. Lainidi et al. (2023) further show that voice and silence can coexist and that speaking-up interventions often achieve disappointing results. Taken together, these studies support the argument that making concerns expressible is not the same as ensuring that organizations recognize and act on them. The proposed capacities offer one explanation for this gap, but the interpretation remains to be tested through comparative and longitudinal research in healthcare and other high-pressure settings.

## 16 Theoretical contributions

The first theoretical contribution is the conceptualization of organizational inaction as a distinct organizational-level construct. Existing destructive leadership research has primarily examined harmful leader behavior, follower susceptibility, and contextual conditions that facilitate destructive influence. The present framework shifts attention toward the organizational processing of destructive leadership after signals of harm have emerged. Organizational inaction is not treated as the mere absence of individual voice or as a residual consequence of ineffective leadership. It refers to a patterned organizational failure to convert available signals into recognition, escalation, and credible intervention. This construct extends destructive leadership research by making organizational response itself an object of theoretical explanation (Einarsen et al., 2007; Padilla et al., 2007; Thoroughgood et al., 2018).

The second contribution is the distinction between recognition capacity and intervention capacity as analytically separate and continuous dimensions. This distinction explains why superficially similar patterns of non-response may result from different organizational deficits. Organizations may possess the authority and governance structures required for intervention while lacking the informational and interpretive capacity to recognize destructive leadership. Conversely, organizations may recognize harmful leadership but remain unable or unwilling to intervene. Crossing both dimensions produces four heuristic ideal types—functional, blind, paralyzed, and incapable organizations—which provide a differentiated framework for theory development and empirical comparison without assuming fixed or mutually exclusive organizational categories (Weber, 1949; Doty and Glick, 1994).

The third contribution is the integration of psychological and organizational mechanisms within a response-oriented framework. Organizational silence and psychological safety explain whether employee experiences become communicable and develop into organizational recognition. Institutional decoupling explains why formal governance structures may fail to generate effective accountability. Normalization of deviance and useful illegality explain how harmful leadership practices may become routine, functionally embedded, and increasingly costly to sanction. By locating these mechanisms within the recognition–intervention framework, the theory connects previously fragmented research streams and specifies how each mechanism contributes to different forms and trajectories of organizational inaction (Morrison and Milliken, 2000; Edmondson, 1999; Meyer and Rowan, 1977; Vaughan, 1999; Kühl, 2019).

Together, these contributions reposition destructive leadership as both a leadership phenomenon and an organizational response problem. The framework does not replace leader-, follower-, or environment-centered explanations. Rather, it extends them by explaining why organizations exposed to comparable forms of destructive leadership may differ systematically in whether harmful conduct becomes visible, actionable, and ultimately sanctionable.

## 17 Practical implications for healthcare organizations

The practical value of the framework lies in distinguishing the organizational capacity that is primarily deficient. Healthcare organizations should not assume that awareness, formal policies, or reporting channels automatically produce effective intervention. Diagnostic and preventive measures should instead be aligned with whether the organization primarily lacks recognition capacity, intervention capacity, or both.

Blind organizations require measures that improve the visibility and interpretation of destructive leadership. Priorities include psychologically safe reporting channels, regular employee feedback, structured leadership diagnostics, multi-source assessments, and escalation procedures that allow weak or fragmented signals to be aggregated across teams and hierarchical levels. Healthcare organizations should also train managers, human resource professionals, and governance actors to distinguish destructive leadership from legitimate performance pressure, interpersonal conflict, or temporarily directive behavior. The objective is not to classify every difficult interaction as destructive, but to improve the organization's capacity to identify repeated and systematic patterns of harm (Morrison and Milliken, 2000; Edmondson, 1999; Frazier et al., 2017).

Paralyzed organizations require a different intervention logic. Their central problem is not the absence of information but the failure to transform recognized harm into credible action. Priorities include clearly assigned decision rights, independent investigation pathways, enforceable sanctions, protection against retaliation, and escalation routes that remain effective when powerful or operationally important leaders are involved. Governance bodies must possess not only formal responsibility but also sufficient authority, information access, and organizational independence. Without these conditions, complaint systems and leadership standards risk remaining symbolic (Meyer and Rowan, 1977; DiMaggio and Powell, 1983).

Incapable organizations require more comprehensive organizational development because both recognition and intervention capacities are weak. Isolated instruments such as a new reporting channel, leadership workshop, or revised policy are unlikely to be sufficient where destructive practices have become culturally and structurally embedded. Such organizations may require simultaneous reform of leadership selection, promotion criteria, employee voice systems, governance structures, accountability mechanisms, and cultural expectations. External oversight or independent diagnostic support may be necessary where internal actors are unable to assess the problem credibly or where destructive leadership has diffused across multiple hierarchical levels.

Functional organizations should focus on maintaining and periodically testing their recognition and intervention capacities. The existence of functioning systems at one point in time does not guarantee their continued effectiveness. Leadership changes, resource scarcity, restructuring, or increased reliance on informal workarounds may gradually weaken psychological safety, reporting credibility, or governance independence. Functional organizations should monitor whether concerns continue to travel upward, whether investigations remain timely and independent, and whether interventions produce organizational learning rather than merely resolving individual cases.

Across all organizational types, healthcare organizations should examine the role of informal workarounds and useful illegality. Informal deviations may be necessary to maintain care continuity under staffing shortages, emergencies, and contradictory requirements. However, organizations should distinguish between temporary professional discretion and dependencies that shield destructive leaders from accountability. Governance reviews should examine not only whether formal rules exist, but also which informal practices sustain daily operations, who controls them, and whether intervention against a destructive leader would expose broader structural weaknesses. This analysis is essential because apparently indispensable leaders may derive their organizational protection from the same gray-zone practices that make intervention difficult (Luhmann, 2022; Kühl, 2019; Straub and Lemmer, 2016).

The framework also provides a practical diagnostic orientation for healthcare management, human resource functions, and supervisory bodies. Organizations should ask two separate questions: are we able to recognize destructive leadership reliably, and are we able to intervene effectively once it has been recognized? These questions should become explicit components of leadership selection, leadership development, governance reviews, workforce strategy, and professional education. Sustainable prevention requires organizational learning across cases, including analysis of how warning signals emerged, where escalation failed, which dependencies delayed intervention, and whether corrective action strengthened the organization's future response capacity (Craig and Gustafson, 1998; Ramos, 2006; Herzka, 2013).

Table 2 summarizes the primary diagnostic deficits, warning signs, and intervention priorities associated with each organizational type.

**Table 2:** Type-specific diagnostic priorities and organizational interventions.

Organizational type	Primary diagnostic deficit	Illustrative warning signs	Priority organizational interventions
Blind	Low recognition capacity despite available intervention structures.	Fragmented or unaggregated complaints; weak upward voice; destructive conduct reframed as toughness, conflict, or performance pressure; superficial leadership diagnostics.	Strengthen psychological safety and reporting channels; aggregate signals across units and hierarchy levels; use regular employee feedback and multi-source leadership assessments; train HR, managers, and governance actors to recognize repeated patterns of harm.
Paralyzed	Recognition exists, but intervention capacity is weak or politically constrained.	Known complaints without consequences; delayed investigations; unclear decision rights; symbolic policies; protection of powerful or operationally important leaders.	Clarify decision rights and escalation routes; establish independent investigations; ensure access to information, protection against retaliation, and enforceable sanctions; strengthen governance authority and independence.
Incapable	Recognition and intervention capacities are both weak.	Destructive practices across several levels; weak selection and promotion systems; normalized misconduct; low trust and voice; ineffective controls; repeated unresolved cases.	Implement coordinated organizational development across leadership selection, promotion, voice systems, culture, governance, and accountability; use independent diagnostic support or external oversight where internal assessment is not credible.
Functional	No primary deficit; risk of gradual erosion of existing capacities.	Declining reporting credibility; slower or less independent investigations; growing resource pressure; increased dependence on informal workarounds; recurring cases without organizational learning.	Periodically test recognition and intervention systems; monitor reporting, investigations, and outcomes; review the effects of leadership changes and resource scarcity; use resolved cases to strengthen organizational learning and prevention.

The organizational types represent heuristic configurations on continuous recognition and intervention dimensions. The diagnostic deficits and illustrative warning signs are theoretically derived from the proposed typology and are intended to guide organizational reflection and future empirical examination rather than to function as validated diagnostic criteria.

## 18 Limitations and future research

The proposed framework has several limitations. First, it is a conceptual reconstruction based on the integration of existing research rather than on a large comparative empirical dataset. The four organizational types are analytically specified but not yet empirically validated as discrete categories. Organizations may display hybrid configurations, and the thresholds separating higher and lower levels of recognition and intervention capacity remain to be determined empirically. Second, the framework emphasizes organizational capacities and structural mechanisms and gives less attention to the micro-level sensemaking processes through which employees and managers interpret, negotiate, and respond to destructive leadership in everyday interactions. Future research should consequently examine both the organizational-level configurations proposed here and the individual and collective interpretations through which these configurations are reproduced or challenged (Flick, 2014; Weick, 1995; Maitlis and Christianson, 2014).

A first priority for future research is the qualitative examination of the proposed mechanisms and organizational types. Semi-structured interviews are particularly suitable because recognition and intervention capacity depend on organizational interpretations, informal knowledge, escalation experiences, power relations, and perceptions of formal and informal practices that may not be visible in standardized organizational data. Interviews should include actors from different organizational positions, such as employees, line managers, senior managers, human resource professionals, and governance or compliance functions. Comparing their accounts would make it possible to examine whether signals of destructive leadership remain localized, how they become organizational knowledge, and why recognized concerns do or do not result in intervention. Table 1 and Supplementary Table 1 outline how the propositions can be translated into interview-based indicators and qualitative examination strategies.

The relational propositions require a research logic that is more comparative than purely illustrative. Researchers should compare organizational cases or units that differ in psychological safety, resource scarcity, hierarchical pressure, governance effectiveness, and reliance on informal workarounds. Such comparisons would allow them to examine whether the proposed mechanisms are systematically associated with differences in recognition and intervention capacity. Future studies should also incorporate negative-case analysis by actively searching for cases that contradict the expected relationships—for example, organizations that recognize destructive leadership despite low psychological safety or that intervene effectively despite severe resource constraints. Interview data should, where possible, be triangulated with organizational documents, complaint procedures, investigation records, employee surveys, or reconstructed critical incidents. This would reduce reliance on single perspectives and permit the consideration of plausible rival explanations.

Longitudinal and retrospective process research is especially important for examining the dynamic propositions of the framework. Proposition 5 assumes that repeated exposure can normalize initially questionable conduct, while Proposition 7 proposes that persistent blindness or paralysis can produce feedback loops leading toward organizational incapability. These processes cannot be evaluated adequately through isolated cross-sectional accounts alone. Future studies should reconstruct sequences of critical incidents, changes in reporting behavior, shifts in trust and voice, organizational responses, and the increasing or decreasing reliance on informal exceptions. Repeated interviews or longitudinal case studies would be particularly valuable for determining whether organizational types remain stable, improve through corrective action, or deteriorate through cumulative non-response (Doty and Glick, 1994; Vaughan, 1999).

Healthcare organizations provide a valuable critical setting for this research program because professional hierarchies, resource constraints, ethical obligations, regulatory requirements, and reliance on informal coordination make tensions between recognition, intervention, and operational continuity particularly visible. Comparative studies across hospitals, residential care facilities, and ambulatory services could examine whether the proposed mechanisms vary across different forms of healthcare organization. However, future research should also extend the framework to other high-pressure and highly regulated fields, such as public administration, emergency services, education, or safety-critical industries. Cross-sector comparison would help distinguish mechanisms that are specific to healthcare from more general patterns of organizational inaction and would clarify the wider applicability of the construct and typology (Weber, 1949; Doty and Glick, 1994).

## 19 Conclusion

Destructive leadership persists not only because some leaders are manipulative, ruthless, or narcissistic. It also persists because organizations differ in their ability to perceive such conduct and to act against it. The study of leadership failure therefore requires a shift in emphasis. Organizational psychology must pay as much attention to structures of inaction as it pays to the psychology of destructive leaders. Only then can the full dynamics of destructive leadership be understood (Einarsen et al., 2007; Thoroughgood et al., 2018).

Empirical examination of the model could support a broader reorientation of destructive leadership research. Future studies could examine organizational inaction across sectors, comparing both the prevalence of destructive leadership and the organizational responses it elicits. Such comparisons would clarify whether recognition and intervention deficits vary systematically across industries and would help tailor interventions to the specific deficit an organization displays (Padilla et al., 2007).

Organizations can contribute to the persistence of destructive leadership through patterned failures of recognition and intervention. In some organizations, harmful conduct remains unrecognized; in others, recognized concerns do not lead to credible action. In still others, destructive leadership becomes intertwined with informal practices that sustain daily operations. This perspective does not excuse destructive leaders or diminish the suffering of affected employees. It locates that suffering within an organizational order whose responses can fail in recurrent and theoretically identifiable ways (Kühl, 2019; Straub and Lemmer, 2016).

## Data Availability

No original data were generated or analyzed for this study. This article presents a conceptual framework based on the sources cited in the manuscript.
